# Multiorgan and Multisystem Involvement During Primary HIV Infection: A Case Report With a Literature Review

**DOI:** 10.7759/cureus.74759

**Published:** 2024-11-29

**Authors:** Gurpremjit J Singh, Deepak Kumar, Ankur Mittal, Vikas Panwar, Harshit Agarwal

**Affiliations:** 1 Surgical Disciplines, All India Institute of Medical Sciences, Rishikesh, Rishikesh, IND; 2 Urology, All India Institute of Medical Sciences, Rishikesh, Rishikesh, IND

**Keywords:** abscess, hiv-infected patients, immunocompromised patient, multiorgan, renal abscess

## Abstract

The initial six months following HIV infection have a high viral load. Nonspecific presentations might lead to the missing primary HIV diagnosis. Multiorgan and multisystem diagnosis is a rare presentation of primary HIV.

A 40-year-old male patient with no documented comorbidities presented with bilateral flank pain. The onset of the pain was gradual, characterized as dull and aching, with radiation toward the groin. The patient also had severe pain in the left shoulder and left ankle region. Per-abdominal examination revealed bilateral flank tenderness, with the right side being more tender than the left. Swelling of the left shoulder and ankle was observed, accompanied by tenderness and a restricted range of motion.

Contrast-enhanced computed tomography of the chest and abdomen revealed bilateral pyelonephritis multiple renal abscesses and liver abscesses. Left shoulder septic arthritis was also present. Pus culture and blood culture were positive for methicillin-sensitive *Staphylococcus aureus*. Antibiotics, according to the culture sensitivity, were given, and retroviral therapy was started from the antiretroviral clinic.

Primary HIV infection can present with a variety of signs and symptoms. This case demonstrates that AIDS can affect any organ and mimic other disease processes. The initial clinical picture might be confused with individually occurring diseases; therefore, we should consider AIDS in patients presenting with multiorgan failure. Early initiation of empiric and then culture-specific antibiotics along with antiretroviral therapy helps in the rapid improvement of the patient and controls the high viremia. The infected patient also needs appropriate counseling on ways to avoid high-risk behavior, which may prevent transmission of HIV.

## Introduction

Primary HIV infection (PHI) in the acute setting presents nonspecific symptoms and complicates the diagnosis [1]. The typical clinical presentation of PHI usually occurs two to four weeks after exposure to the virus and most commonly presents as fever, pharyngitis, and adenopathy. Therefore, the early diagnosis might be missed by the clinical picture, given these nonspecific symptoms [2]. Rapid diagnosis of PHI prevents unknowing virus transmission during periods of high viral load [1]. Establishing an early diagnosis is crucial for the individual and public health. Herein, we present a case of PHI with multiorgan and multisystem involvement. This article was previously presented at the 2021 Urological Society of India Annual Conference on February 12, 2021.

## Case presentation

A 40-year-old male patient with no documented comorbidities presented with bilateral flank pain. The onset of the pain was gradual, characterized as dull and aching, with radiation toward the groin. The patient also had severe pain in the left shoulder and ankle region. There was a history of occasional smoking and drinking. There was no history of fever, chills, hematuria, previous surgery, respiratory complaints, or decreased urine output. The patient and relatives denied any history of illicit drug abuse.

General physical examinations were within normal limits. Per-abdominal investigation revealed bilateral flank tenderness, more on the right than on the left. Left shoulder and ankle swelling were present, with tenderness and a restricted range of motion. No apparent enlarged lymph nodes were present.

Blood tests showed hemoglobin of 7.3 g/dL and a total leukocyte count of 5,200/mm^3^. Liver and kidney function tests were within normal limits. Hepatitis B surface antigen and hepatitis C virus serology were negative. However, HIV serology came to be positive. On further evaluation, the absolute CD4 count was 68 cells/mm^3^, and the percentage of T helper cells CD4 was 9.2%, respectively.

**Figure 1:**
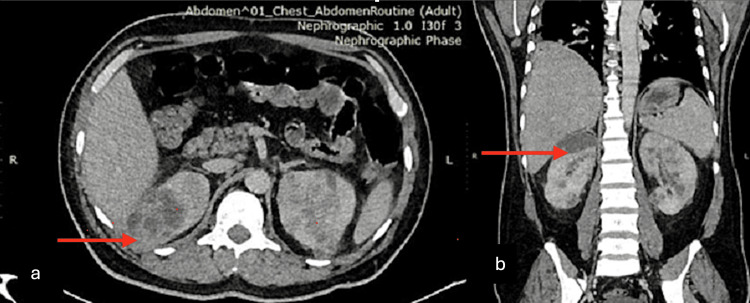
CECT abdomen. Axial (a) and coronal (b) images showing right upper pole renal abscess and multiple other renal abscesses in bilateral kidneys (arrows represent the areas of abscesses) CECT: contrast-enhanced computed tomography

**Figure 2:**
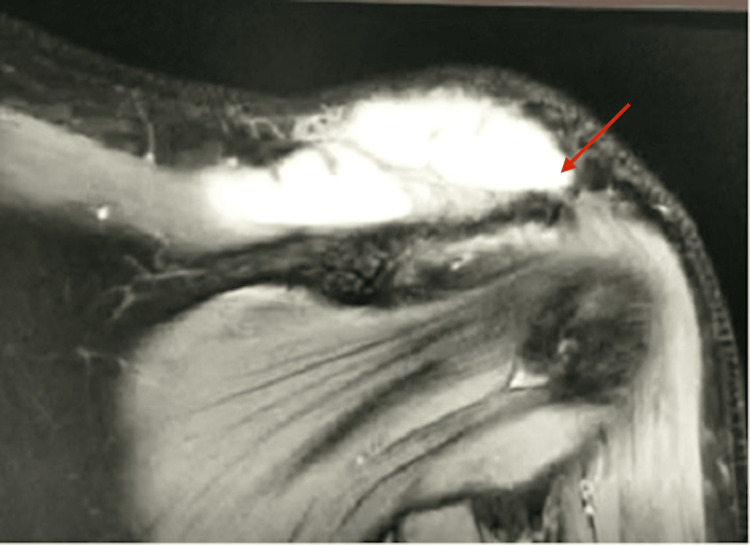
MRI of the left shoulder, showing an irregular collection in the left acromioclavicular joint (arrow represents the area of collection)

Contrast-enhanced computed tomography of the chest and abdomen revealed bilateral pyelonephritis and multiple renal abscesses with the largest size, 7.2 x 4.3 x 2.4 cm, at the upper pole of the right kidney (Figure 1). It also showed an enlarged liver (23.4 cm in craniocaudal) with few peripherally enhancing hypodense lesions suggestive of abscesses and mild right-sided pleural with fissural extension with underlying basal atelectasis. Ultrasound of the left shoulder joint depicted an ill-defined heteroechoic collection with free-floating internal echoes in the acromioclavicular joint, causing significant joint space widening. The left ankle showed multifocal ill-defined hypoechoic intramuscular groups with internal echoes involving the deep muscles of the ankle, the largest measuring 10 x 5 cm along the medial aspect. MRI was done for the left shoulder and ankle, which further revealed irregular multiloculated peripherally enhancing collections measuring 7.7 x 9.1 x 3.3 cm with epicenter at the acromioclavicular joint with superior extension up to the subcutaneous plane, causing a focal bulge (Figure 2). Inferior-medial extension into the intramuscular plane between the trapezius and supraspinatus muscle and anteroinferior extension into the clavicular part of the deltoid muscle were also noticed.

The patient was started on broad-spectrum antibiotics. USG-guided aspiration of pus from the right renal abscess, liver abscess, and left shoulder joint was done. Pus culture and blood culture were positive for methicillin-sensitive *Staphylococcus aureus* sensitive to amoxiclav, tetracycline, linezolid, and vancomycin. Tuberculosis acid-fast bacillus and cartridge-based nucleic acid amplification tests were negative. Fungal culture was also negative. Repeat aspiration of residual abscesses in the upper pole of the right kidney and left ankle joint was done. Vancomycin was started empirically, which was continued following the culture sensitivity report. Antiretroviral therapy (ART) was started with a fixed drug combination of tenofovir /lamivudine/dolutegravir. Echocardiography was also done, which showed a normal study. No vegetation was found. A prophylactic antibiotic (trimethoprim-sulfamethoxazole combination) was created because of a low CD4 count. After aspiration, with culture-sensitive antibiotics and ART, the patient continued to improve and was discharged on day 12. The patient is on regular follow-ups in the ART clinic, orthopedics, and urology.

## Discussion

The first six months following HIV exposure is the PHI period [3]. The initial six months have a high viral load, which the host immune system subsequently limits. Without ART, there is a gradual decline in CD4 T cells. Viral dissemination is rapid in this period, and viral reservoirs form throughout the body. This is why ART cannot cure HIV infection [3]. Symptoms take two to six weeks to appear.

The presentations of primary HIV can be the following:

1) the most common is a flu-like illness, including fever, cough, lymphadenopathy, and myalgias [4], which occurs in 65%-95%;

2) neurological presentations, including aseptic meningitis and facial nerve paresis [4];

3) multiorgan involvement, including renal infarcts, renal failure, myocarditis, and pancreatitis [2];

4) multiorgan abscess and septic arthritis as in this index case.

The acute presentations may be nonspecific, making the diagnosis challenging [1]. A delay in diagnosis results in a higher rate of disease progression and rapid fall in CD4 T cells. This also increases the chances of opportunistic infections. The Strategic Timing of the Antiretroviral Treatment trial concluded that immediate initiation of ART benefits all patients [5].

PHI can have a bizarre clinical presentation. Paño-Pardo et al. presented a case of a 19-year-old female patient with PHI with an unusual presentation of fever, rhabdomyolysis, myocarditis, pancreatitis, bilateral renal infarcts, anemia, and acute kidney injury. The patient improved on antibiotics [2]. Tattevin et al. reported a case of a 16-year-old woman with an unusual presentation of sepsis, leukopenia, myocarditis, and rhabdomyolysis during PHI [6].

This index patient presented with multiorgan and multisystem abscesses. Renal and liver abscesses with septic arthritis of the left acromioclavicular joint and left ankle were seen in the index patient and managed. The entire presentation may be due to PHI, as no other predisposing factors existed. *S. aureus* bacteremia has a higher incidence among patients with HIV. The risk is related to the CD4+T cell counts and is highest in patients with CD4 counts <200 cells/mm^3^. Bacteremia can be classified as complicated or noncomplicated, particularly in patients with metastatic sites of infection, as was present in this index patient having both renal and liver abscesses. Patients may also present with persistent systemic inflammatory response syndrome and cardiovascular dysfunction [7].

Patients who develop AIDS are at increased risk of unusual kidney infections with agents like Candida, Mycobacteria, Salmonella, and Pneumocystis pneumonia. Renal Candida infections can result in fungal ball formation in the collecting system, leading to hydronephrosis. A staphylococcal renal abscess can also be present and repeatedly occur, leading to nephrectomy. Renal tuberculosis can also be seen in 6%-23% of cases of patients with HIV and may require antituberculosis agents for six to nine months. Therefore, the patient should undergo testing for tuberculosis and fungal infections to rule out additional conditions [8].

A retrospective analysis of 18 patients with septic arthritis with HIV showed that three patients in their study were diagnosed with HIV after admission for septic arthritis. The mean CD4+T cell count was 154/mm^3^. *S. aureus* cultures were present in seven patients, streptococcal in six patients, coagulase-negative Staphylococcus in two patients, *Neisseria gonorrhoeae* in one patient, and *Mycobacterium tuberculosis* in three patients. The culture was monomicrobial in 17 patients and polymicrobial in one patient. Fever was present in 18 patients in this study. Three patients who had adjacent osteomyelitis with septic arthritis underwent debridement and drainage procedures [9]. The index patient in this study had polyarticular septic arthritis, which should also be suspected in patients having a CD4+T cell count of less than 200 cells/mm^3^. The patient also had cultures positive for *S. aureus*.

These microbiological data show that *S. aureus* is a common pathogen in HIV patients and should always be covered in initial empiric antibiotic therapy. Although the index patient in this study has methicillin-sensitive *S. aureus*, many studies have reported an increased incidence of methicillin-resistant *S. aureus* in HIV-infected patients [10,11]. A high index of suspicion should be present for opportunistic pathogens. Therefore, any purulent aspirated material should be sent for mycobacterial and fungal cultures. This is necessary for patients having a CD4+T cell count of less than 200 cells/mm^3^ [9].

Early diagnosis is crucial to prevent virus transmission to uninfected sexual partners. Knowledge of HIV also limits transmission with more frequent use of condoms. The principle of treatment-as-prevention refers to using ART to reduce the viral load and decrease the chances of transmission [12].

## Conclusions

PHI can present with a variety of signs and symptoms. It should be kept in the differential diagnosis of patients with multiorgan and multisystem involvement. Primary HIV should be differential for such presentation besides tuberculosis, autoimmune pathology, etc. Early initiation of broad-spectrum empiric (based on local hospital antibiotic policy) and then culture-specific antibiotics along with ART helps in the rapid improvement of the patient and controls the high viremia. The infected patient also needs appropriate counseling on ways to avoid high-risk behavior, which may prevent the transmission of HIV.
